# Histone Methylation by NUE, a Novel Nuclear Effector of the Intracellular Pathogen *Chlamydia trachomatis*

**DOI:** 10.1371/journal.ppat.1000995

**Published:** 2010-07-15

**Authors:** Meghan E. Pennini, Stéphanie Perrinet, Alice Dautry-Varsat, Agathe Subtil

**Affiliations:** 1 Institut Pasteur, Unité de Biologie des Interactions Cellulaires, Paris, France; 2 CNRS URA 2582, Paris, France; Duke University, United States of America

## Abstract

Sequence analysis of the genome of the strict intracellular pathogen *Chlamydia trachomatis* revealed the presence of a SET domain containing protein, proteins that primarily function as histone methyltransferases. In these studies, we demonstrated secretion of this protein via a type III secretion mechanism. During infection, the protein is translocated to the host cell nucleus and associates with chromatin. We therefore named the protein nuclear effector (NUE). Expression of NUE in mammalian cells by transfection reconstituted nuclear targeting and chromatin association. *In vitro* methylation assays confirmed NUE is a histone methyltransferase that targets histones H2B, H3 and H4 and itself (automethylation). Mutants deficient in automethylation demonstrated diminished activity towards histones suggesting automethylation functions to enhance enzymatic activity. Thus, NUE is secreted by *Chlamydia*, translocates to the host cell nucleus and has enzymatic activity towards eukaryotic substrates. This work is the first description of a bacterial effector that directly targets mammalian histones.

## Author Summary

*Chlamydia trachomatis* is a particularly prevalent human pathogen responsible for loss of eyesight through trachoma and is the most common sexually transmitted disease of bacterial origin. Unlike most other bacterial pathogens, chlamydiae survive only within another cell and thus must develop sophisticated mechanisms to subvert immune clearance by their host. To this end, the bacteria secrete proteins into the cells they occupy which disrupt normal cellular function. In this work, we identify one such protein, NUE, which is injected into the nucleus of human cells during infection. Sequence analysis of NUE revealed the presence of a SET domain which suggests its involvement in chromatin remodeling. Indeed, we found the protein associated with chromatin both during infection and when transfected into human cells. Importantly, we demonstrate NUE is an active histone methyltransferase that targets host cell histones but does not modify bacterial histone-like proteins. This is the first bacterial protein identified which is able to penetrate the nucleus and directly modify mammalian histones.

## Introduction

*Chlamydiae spp.* are responsible for a variety of significant diseases in both animals and humans. *Chlamydia trachomatis* is the most prevalent sexually transmitted bacterial pathogen, infecting an estimated 92 million people a year, and leads to severe pathologies including infertility, ectopic pregnancy and pelvic inflammatory disease. Additionally, *C. trachomatis* infection of the ocular epithelium is the leading cause of blindness by an infectious agent and *C. pneumoniae* is a prevalent respiratory pathogen that has been implicated in coronary artery diseases [1].

Chlamydiae are obligate intracellular pathogens that target epithelial cells and have a specific biphasic developmental cycle. The infectious form of the bacteria, called elementary bodies (EBs), are characterized by a rigid cell wall, densely packed DNA and metabolic inactivity. Upon entry of a host cell, EBs rapidly convert to reticulate bodies (RBs), the metabolically active but noninfectious form of the bacteria. RBs replicate within a membrane-bound vacuole in the host cell called an inclusion. The bacteria remain within inclusions until they eventually convert back to infectious EBs and exit the host cell as a result of cell lysis or via fusion of the inclusion with the cell membrane [2].

Like other pathogenic gram negative bacteria, *C. trachomatis* encodes a type three secretion (TTS) system that enables the translocation of proteins across a eukaryotic host membrane. In the case of chlamydiae, TTS occurs both across the plasma membrane during entry and across the inclusion membrane during the intracellular developmental cycle. There are no common signal sequences found in proteins secreted by TTS although it is generally accepted that the signal is located at the N-terminus [3]. It is therefore infeasible to identify effector proteins by sequence alone. As chlamydiae are intractable for genetic manipulation, it is also not possible to identify secreted proteins using bacterial mutants. Despite these experimental obstacles, several groups have identified chlamydial proteins secreted by TTS [4], [5], [6], [7] Although the specific function of most of these proteins remains unknown, they presumably target various cellular processes and allow the bacteria to subvert host defense mechanisms. To date, no such effectors have been found to target the host cell nucleus.

The SET domain is a 130-residue domain originally defined in proteins capable of changing the expression of heterochromatin-embedded gene sequences [8]. Subsequent studies identified these proteins as histone methyltransferases (HMTs) whose enzymatic function is covalent attachment of methyl groups to lysine residues of histones. A SET domain containing protein has been identified in every eukaryote studied [9], yet these proteins are notably underrepresented in prokaryotes presumably because they lack the target substrate, histones. The majority of non-eukaryotic SET domain proteins are found in species that interact with eukaryotes, such as pathogenic bacteria or viruses. Although these enzymes are generally presented as specific for one particular histone modification, several reports have found multiple histone and/or non-histone cellular substrates for a single SET domain protein [10], [11], [12]. In some cases, this includes methylation of more than one histone or methylation of multiple residues within a particular histone [13], [14], [15]. Regardless of the substrate, all SET domain proteins require the presence of S-adenosyl-L-methionine (SAM) as the methyl donor.

Eukaryotic DNA is tightly packaged in the nucleus as nucleosomes, a repeating unit of 146 bp of DNA encircling an octamer of core histones (two copies each of H2A, H2B, H3 and H4). Higher order chromatin structure is regulated in part by N-terminal histone tails of the core histones that protrude from the nucleosome and are available for inter-nucleosomal interactions. These histone tails are the target of various post-translational modifications including acetylation, phosphorylation, ubiquitination and methylation. Each of these modifications contributes to reduced or enhanced accessibility of the transcriptional machinery at a particular locus and ultimately dictates whether a gene is expressed or repressed. The biological consequence of histone methylation varies greatly depending on the targeted histone, the specific lysine modified and the number of methyl groups added to this lysine (mono-, di- or tri-methylation). The effects of these modifications range from gene suppression to gene activation, either directly due to a change in chromatin structure or indirectly through the recruitment of additional modifying proteins [9], [16]. Among histone modifications, methylation is thought to be a relatively stable modification resulting in longterm changes in cellular function. Once thought to be a permanent modification, the growing number of histone demethylases being identified suggests that histone methylation is a dynamic process [17], [18].

Unlike eukaryotes, prokaryotes do not have histones nor highly ordered chromatin. However, several reports have identified histone-like proteins in bacteria [19]. The specific function of these proteins remains largely undefined. *Chlamydia trachomatis* encodes two histone-like proteins called histone H1-like chlamydial protein 1 and 2, or Hc1 and Hc2. Both are thought to play a critical role in compacting bacterial DNA during the transition from RBs to EBs [20], [21], [22]. The metabolic inactivity of EBs due to extreme condensation of DNA is thought to be regulated by tight interactions with Hc1 and Hc2. In support of this hypothesis, Hc1 expression is decreased during the EB to RB transition which corresponds temporally to the onset of transcriptional activity in the bacteria [23].

Pathogenic bacteria employ a wide range of strategies to avoid elimination by their host. Targeting histone modifications could allow a pathogen to inhibit transcriptional activation of host defense genes. In this study, we set out to characterize the chlamydial SET domain protein CT737. We found the protein to be secreted from bacteria and localized to the mammalian nucleus. Additionally, we report its enzymatic activity as a histone methyltransferase. Altogether, we propose to designate CT737 and its homologs in other chlamydiae genomes as NUE, as it is the first nuclear effector identified in chlamydiae.

## Results

### NUE contains a conserved SET domain

**Figure 1:**
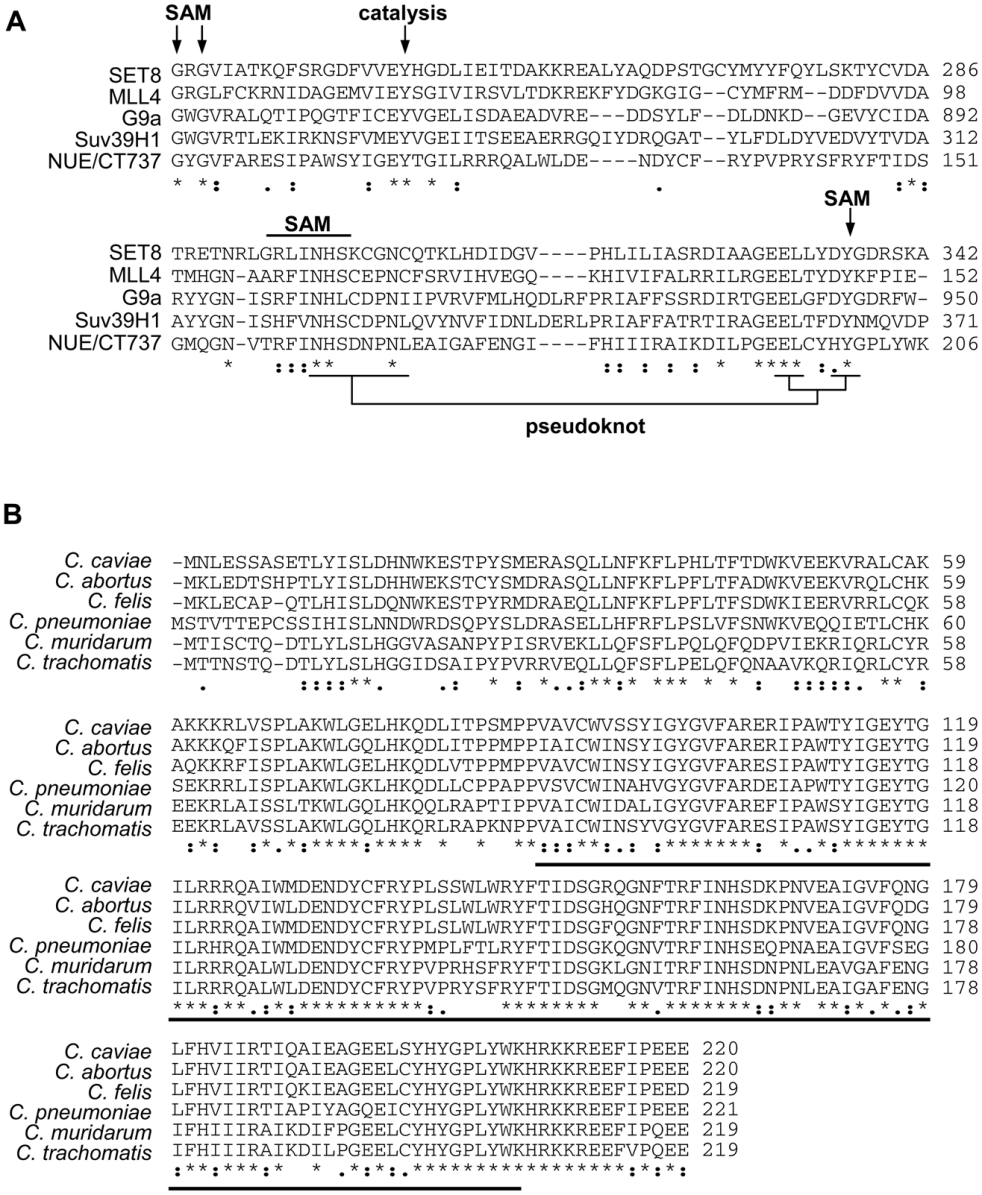
Sequence analysis of chlamydial SET domain protein. (A) The SET domain of chlamydial protein NUE (CT737) sequence was compared to eukaryotic SET domain proteins G9a, MLL4, Set8 and Suv39H1. Residues important for SAM binding, catalysis and the structurally important pseudoknot are noted. (B) Homologous SET domain proteins were compared for each of the 6 Chlamydia genomes sequenced, the SET domain is underlined. “.” indicates a semi-conserved residue, “:” a conserved residue and “*” identical residues in all sequences.

Genomic analysis is a highly useful tool when studying protein function of a genetically intractable pathogen such as chlamydiae. Completion of the *Chlamydia trachomatis* genome sequence revealed a SET domain in ORF 737 [24]. We analyzed the similarity of CT737 (NUE) to a select number of mammalian SET domain proteins (Fig. 1A). Within the predicted enzymatic portion of the protein, we found between 14% and 28% sequence identity with eukaryotic SET domain proteins, which is within the range of similarity found among mammalian SET domain proteins themselves. Importantly, the residues critical for enzymatic function are highly conserved. This includes the SAM binding site, the site of catalysis and the unique pseudoknot structure required for enzyme function [25]. Additionally, we compared the sequences of all six sequenced chlamydiae species, including the two human pathogens *C. trachomatis* and *C. pneumoniae*. We found a highly conserved homolog of CT737 in each (Fig. 1B), suggesting that this protein plays an essential role in chlamydiae development.

### NUE is secreted from the bacteria to the host cell nucleus

**Figure 2:**
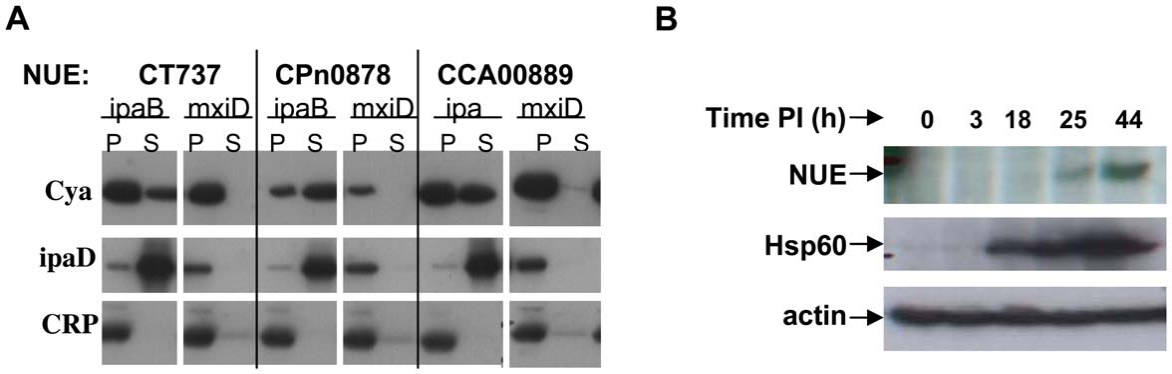
NUE is secreted from bacteria and detected during late infection. (A) *Chlamydia trachomatis* NUE and its *C. pneumoniae* and *C. caviae* homologs were fused to the Cya reporter protein and expressed in *Shigella flexneri* ipaD (constitutive TTS) or mixD (defective TSS) strains. Bacteria were pelleted and both pellet and supernatant were loaded on SDS-PAGE gels, transferred to a PVDF membrane and probed with anti- Cya (to detect chlamydial fusion proteins), anti-IpaD (Shigella secreted protein) or anti-CRP (Shigella non-secreted protein) antibodies. (B) HeLa cells were infected with *C. trachomatis* for the times indicated, lysed and analyzed by Western blot using antibodies for the chlamydial proteins NUE and Hsp60. Actin was used to ensure equal loading of samples. All results shown are representative of at least 2 separate experiments.

Although chlamydiae are evolutionary distant from other gram negative bacteria, we have successfully used a heterologous machinery, *i.e.* the TTS system of *Shigella flexneri* to identify chlamydial TTS substrates [4]. To test the hypothesis that NUE might be a TTS effector protein, we fused the N-terminal sequence of NUE to the reporter protein, calmodulin-dependent adenylate cyclase (Cya). The chimeric protein was then expressed in *S. flexneri ipaB* (constitutive TTS) or *mxiD* (deficient in TTS) strains. When we analyzed the culture supernatant versus the bacterial pellet, we found NUE primarily in the supernatant of *ipaB* culture (Fig. 2A). The same expression pattern was observed for the endogenous TTS substrate of *Shigella*, invasion plasmid antigen D (IpaD). Conversely, we found cAMP receptor protein (CRP), a non-secreted protein retained in *S. flexneri*, exclusively in the bacterial pellet excluding the possibility of non-specific leaking into the supernatant. This result demonstrates the NUE/Cya chimera was secreted to the culture supernantant by the *ipaB* strain. Furthermore, the chimera was found primarily in the bacterial pellet when expressed in the *mxiD* strain, indicating secretion occurs via TTS. Despite the impressive degree of NUE sequence identity among chlamydiae (Fig. 1B), there is little conservation in the N-terminal domain where the secretion signal is located. To determine whether the SET domain protein of other species also contained a TTS signal, we cloned the N-terminal sequence of the NUE *C. pneumoniae* and *C. caviae* homologs (Cpn0878 and CCA00889, respectively) upstream of the Cya reporter. Both chimeras were secreted when expressed in TTS constitutive but not TTS deficient *S. flexneri* (Fig. 2A). Due to the poor conservation of the N-terminal sequence of NUE homologs, finding three sequences that function as TTS signal is highly indicative that chlamydiae SET domains proteins are TTS effectors.

To determine the expression pattern of NUE during infection, we generated an antibody specific for the protein (see Supplementary Figure S1). Using this antibody for immunofluorescence analysis, we were able to detect NUE overexpression in transfected cells but were not able to detect the expression of NUE during infection most likely due to a low level of expression (data not shown). However, we were able to detect NUE in the lysates of infected cells by Western blot (Fig. 2B). In these studies, we infected HeLa cells with *C. trachomatis* for 3–44 h, lysed the cells and probed for NUE. NUE expression is first detectable at late timepoints (between 18 and 25 h). This is in agreement with transcriptome studies describing peak mRNA expression at 18 h post-infection [26]. By comparison, we found expression of chlamydial heat shock protein 60 (Hsp60) at earlier timepoints as expected of chlamydial proteins expressed earlier in infection. While these data suggest that NUE expression occurs late in infection, we cannot rule out the possibility that expression of NUE occurs at earlier timepoints but is undetected due to low abundance.

### Transfected NUE localizes to the nucleus of mammalian cells

**Figure 3:**
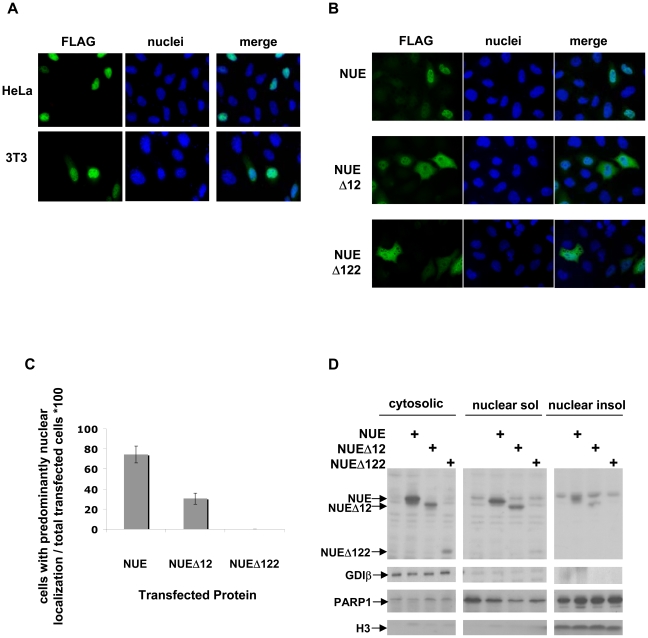
NUE localizes to the host cell nucleus. (A) HeLa or 3T3 cells were transfected with FLAG-NUE, fixed with paraformaldehyde, permeabilized and analyzed by immunofluorescence using anti-FLAG antibody and Hoescht dye to stain nuclei. (B) HeLa cells were transfected with NUE or the C-terminal truncation mutants NUEΔ12 and NUEΔ122 and treated as above for immunofluorescence analysis. (C) The number of transfected cells demonstrating predominant nuclear localization was divided by the number of total transfected cells and multiplied by 100 to determine % nuclear localization. Each experiment was performed 3 times with >500 transfection events counted for each protein. Error bars reflect the difference in calculated percentage among the 3 separate experiments. (D) Western blot analysis of transfected cells. HeLa cells were transfected with FLAG-tagged NUE, NUEΔ12 or NUEΔ122 for 24 h and cells were then fractionated into cytosolic and nuclear fractions. Protein concentration was measured by Bradford and for each fraction 20 µg of protein was loaded on a gel. The remaining pellet after protein extraction from nuclei was also analyzed as the nuclear insoluble fraction. NUE distribution in the different fractions was analyzed by Western blot using anti-FLAG antibody. Anti-GDIβ (cytosolic), anti-PARP1 (nuclear) and anti-H3 (nuclear insoluble) were used to demonstrate purity of the fractions.

Due to the limitations of working with *Chlamydia* itself, we generated both N-terminus FLAG- and GFP-tagged NUE constructs. We found that, regardless of the tag, NUE localized to the nucleus of transfected cells, both murine and human (Fig. 3A, GFP data not shown). Sequence analysis of the protein revealed two potential nuclear localization sequences (RRR at position 121 and KHRKKR at position 206). We therefore generated two C-terminal truncation mutants of NUE, NUEΔ12 and NUEΔ122 that exclude either one (NUEΔ12) or both (NUEΔ122) of these sequences. When HeLa cells were transfected with the FLAG-tagged truncated proteins, both demonstrated a loss of predominant nuclear localization compared to NUE but to varying degrees (Fig. 3B and C). NUEΔ122 completely lost exclusive nuclear localization (0% exclusive nuclear localization) while NUEΔ12 had an intermediate phenotype (30% of transfected cells demonstrated predominant nuclear localization compared to 74% of full-length NUE). Both mutants are expressed diffusely throughout the cell, including in the nuclear space (confirmed by Z-stack analysis, data not shown). Full-length FLAG-NUE has a molecular weight of 28 kDa and could potentially diffuse into the nucleus unassisted. However, the loss of nuclear enrichment with truncated mutants verifies that NUE nuclear localization cannot be solely attributed to low molecular weight. Additionally, we fractionated the transfected cells to probe cytosolic versus nuclear soluble and nuclear insoluble fractions (Fig. 3D). We found, in agreement with the immunofluorescence data, that NUEΔ122 was poorly retained in the nucleus. NUE and NUEΔ12 appeared equally retained in the soluble nuclear fraction. Interestingly, while both NUE and NUEΔ12 were detected in the nuclear insoluble, the full-length NUE showed slightly higher abundance in this fraction. To control for the purity of the fractions, we reprobed the membrane for Rab-GDP dissociation inhibitor β (GDIβ, found only in the cytosolic fractions), poly (ADP-ribose) polymerase 1 (PARP1, found only in the nuclear fractions) and histone H3 (found only in the nuclear insoluble fractions). Taken together, we conclude that while the last 12 amino acids take part in NUE distribution to the nucleus, other internal features such as an internal nuclear localization signal or the SET domain itself, are also required for NUE accumulation in the nucleus.

### NUE is translocated to the host cell nucleus during infection

**Figure 4:**
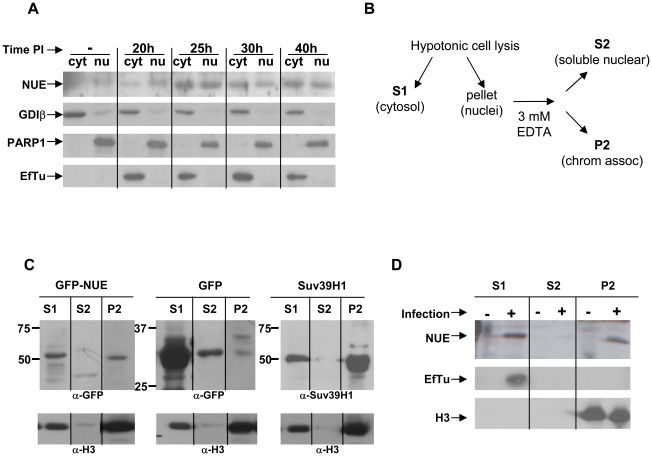
NUE is found in the nucleus of infected cells and associates with chromatin. (A) HeLa cells were infected with *C. trachomatis* for 20, 25, 30 or 40 h. Nuclei were isolated as described in the Method section and both cytosolic and nuclear fractions were analyzed by Western blot using anti-NUE antibody. The membrane was stripped and re-probed for GDIβ as a cytosolic marker, PARP1 as a nuclear marker and the chlamydial protein EF-Tu to ensure the absence of bacteria in the nuclear fraction. Blots are representative of 2 separate experiments. (B) A schematic of the extraction protocol used to detect chromatin-associated proteins. (C) HeLa cells were transfected with GFP-NUE or GFP only as a negative control. Nuclei were separated from the cytosolic fraction of transfected HeLa cells. The nuclei were resuspended in 3 mM EDTA to extract soluble nuclear proteins versus chromatin-associated proteins. Samples were analyzed by Western blot using anti-GFP. HeLa cells transfected with Suv39H1 were used as a positive control and anti-Suv39H1 was used to detect the protein. Histone H3 was used to ensure chromatin enrichment in our chromatin fraction. Blots are representative of 3 separate experiments. (D) HeLa cells were infected with *C. trachomatis* for 40 h and analyzed for chromatin-association of NUE as in (C). Histone H3 was used to demonstrate purity of our chromatin-associated fractions and EF-Tu was used to control for bacterial contamination. Blots are representative of 2 separate experiments.

To determine the localization of NUE during infection, we infected HeLa cells for 20, 25, 30 or 40 h and isolated nuclei by hypotonic cell lysis followed by low speed centrifugation. When we probed the cytosolic (including bacteria) and nuclear fractions by Western blot, NUE was clearly found in the nucleus of infected cells, particularly at late timepoints (Fig. 4A). In contrast, the chlamydial proteins EF-Tu (an abundant protein retained in the bacteria) was absent in our nuclear fractions excluding the possibility that these fractions were contaminated with bacteria. As expected, we found NUE (as well as EF-Tu) in the cytosolic fraction, presumably at a stage of synthesis and secretion. To control for the purity of our fractions, we reprobed the membrane for GDIβ (found only in the cytosolic fractions) and PARP1 (found only in the nuclear fractions). In tandem with our previous results demonstrating the presence of a TTS signal in NUE (Fig. 2A), we conclude that NUE is secreted via TTS and translocated to the nucleus of host cells.

To further characterize the nuclear localization of NUE, we transfected cells with GFP-NUE for 24 h, separated out the nuclei and then fractionated the nuclei into soluble versus chromatin-associated protein as diagrammed in Fig 4B. We found NUE in both the cytosolic and nuclear fractions, as supported by our immunofluorescence data. Nuclear NUE was found in the chromatin fraction (Fig. 4C). As a positive control, we found similar distribution of myc-tagged Suv39H1, a well-characterized histone methyltransferase. In contrast, GFP was found in the cytosolic and soluble nuclear fractions but very little to none was found in the chromatin fraction demonstrating that this association is specific to NUE. We reprobed our membrane with anti-histone H3 to demonstrate histone enrichment in our chromatin fraction and which served as an equal loading control. We conclude that NUE is localized to the nucleus and associated with chromatin, supporting our hypothesis that it plays a role in chromatin function. To address whether NUE is found associated with chromatin during infection, we infected HeLa cells with *C. trachomatis* for 40 h and fractionated the cells into cytosolic, nuclear soluble and chromatin fractions as was done in the transfected cells. Importantly, we found NUE is associated with chromatin in infected cells (Fig. 4D). The absence of EF-Tu confirms this signal is not due to bacterial contamination of the chromatin-associated fraction.

### NUE is a functional histone methyltransferase

**Figure 5:**
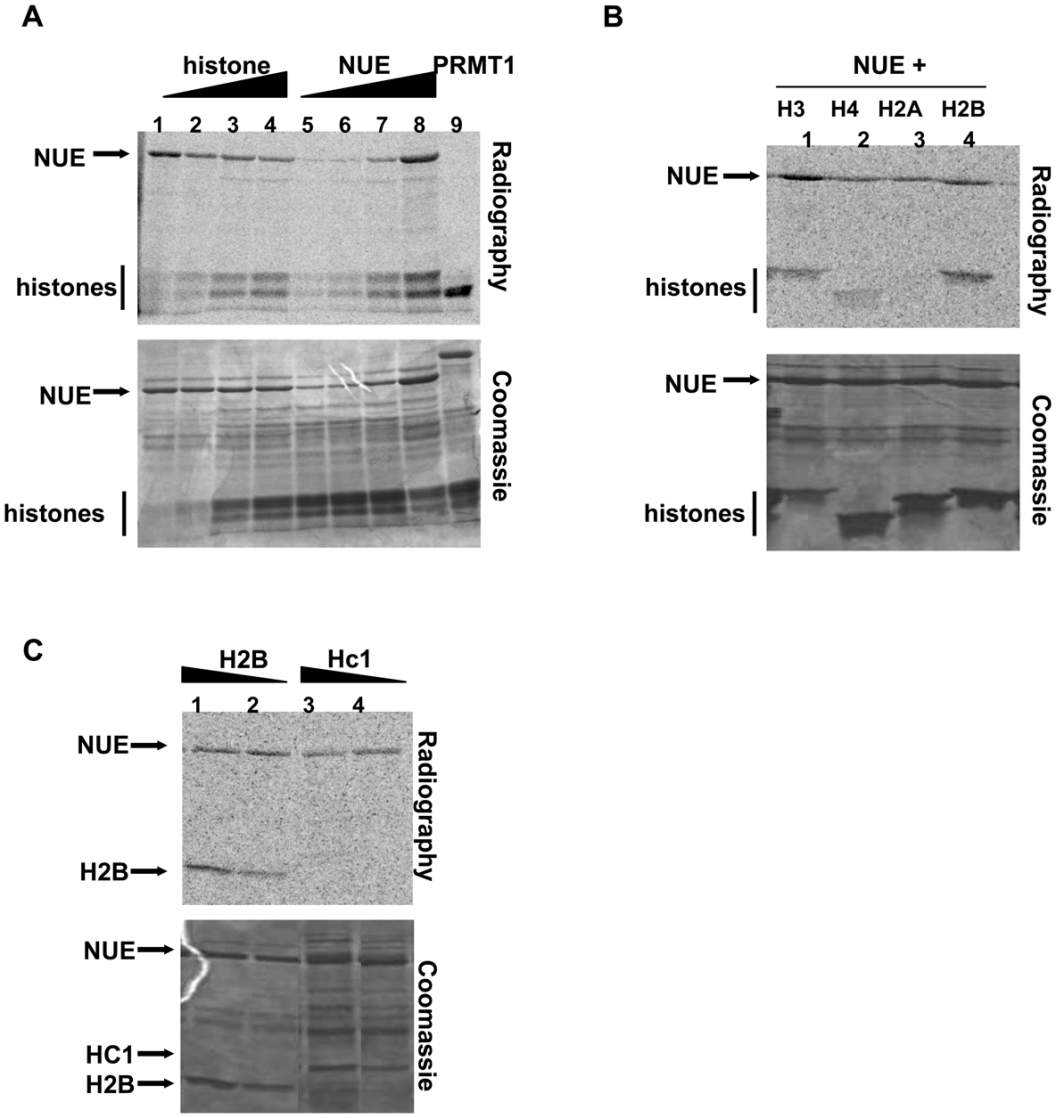
NUE methylates mammalian histones *in vitro*. (A–C) Recombinant GST-NUE was purified from *E. coli* and incubated with either core histones (A), individual recombinant histones (B) or recombinant His-tagged chlamydial protein Hc1 (C) in the presence of ^14^C-SAM for 1 h at 30°C. Samples were then separated on a 15% SDS-PAGE gel and stained with Coomassie blue prior to gel dehydration and 24 h exposure to capture radioactive events. PRMT1, a histone H4 methyltransferase, was used as a positive control in panel A. Various concentrations of histone H2B were used as a positive control in panel C.

SET domain proteins methylate histones using S-adenosyl-L-methionine (SAM) as a methyl donor. In order to determine the enzymatic activity of NUE, we performed *in vitro* methyltransferase assays using GST-NUE purified from *E. coli* and mammalian histones as a potential substrate. We found that NUE methylated multiple core histones (Fig. 5A and B). NUE-mediated methylation is dependent both on the amount of histone substrate (Fig. 5A, lanes 1–4) and on the concentration of enzyme (Fig. 5A, lanes 5–8). As a positive control, we used the histone H4 methyltransferase protein arginine methyltransferase I (PRMT1) (Fig. 5A, lane 9). To assess the individual histones modified by NUE, we repeated the assay using individual recombinant histones. NUE methylated H2B, H3 and to a lesser extent H4, but not H2A (Fig. 5B). Clearly, NUE has a stronger activity towards H4 when all core histones are present as in Fig. 5A. The preference of HMTs for one form of histone over another (*i.e.*, isolated histones versus recombinant) has been reported and is presumably due to slight variations in proteins expressed in mammalian versus bacterial cells [27], [28].

The histone-like chlamydial protein Hc1 plays a role in DNA condensation during the bacterial life cycle. To test the possibility that Hc1 is a substrate for NUE, we conducted *in vitro* methylation assays using various concentrations of purified recombinant Hc1 in the presence of NUE. NUE did not methylate Hc1 at any concentration tested (Fig. 5C). Due to the difficulties in growing and purifying Hc1, we were not able to test concentrations in molar excess of mammalian histones. However, using H2B as our histone control, we clearly demonstrated the preference of NUE for mammalian histones over chlamydial Hc1 when used at equal concentrations.

### NUE automethylates

**Figure 6:**
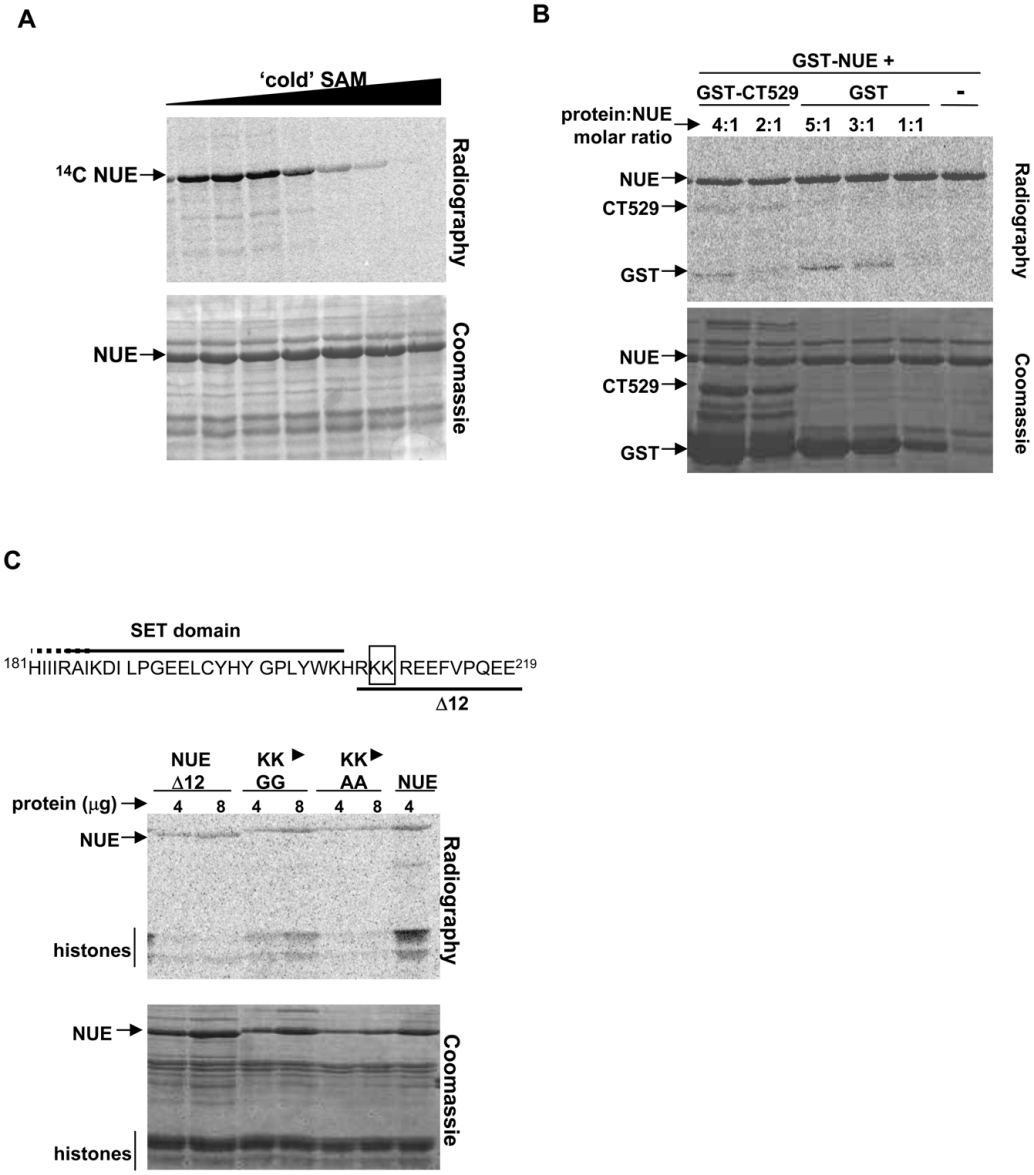
NUE automethylation regulates its activity towards histones. (A) NUE automethylation is SAM-dependent. Recombinant GST-NUE was incubated in the presence of ^14^C-SAM and increasing amounts of non-radioactive SAM for 1 h at 30°C. Samples were then separated by SDS-PAGE electrophoresis and stained with Coomassie blue prior to gel dehydration and 24 h exposure to capture radioactive events. (B) NUE automethylates outside the GST moiety. GST-NUE was incubated with chlamydial protein GST-CT529 (1∶4 or 1∶2 molar ratio) or GST alone (1∶5, 1∶3 or 1∶1 molar ratio). Samples were then treated as in panel A. (C) NUE mutants have diminished activity towards histones. NUE, NUEΔ12, NUEGG (KK→GG) or NUEAA (KK→AA) were incubated in the presence of core histones for 1 h at 30°C. Samples were then treated as in panel A. All gels are representative of at least 2 independent experiments.

While conducting our *in vitro* methylation assays, we consistently detected strong methylation of NUE itself (automethylation). As the amount of histone substrate increased, the level of automethylation decreased (Fig. 5A) suggesting competition between substrates. To confirm this was a *bona fide* methylation event, we performed the assay using excess amounts of non-radioactive SAM to compete away the incorporation of ^14^C-labelled SAM-derived methyl groups (Fig. 6A). Additionally, we wanted to ensure that the automethylation signal of NUE was not solely due to methylation of the GST moiety. We therefore performed *in vitro* methylation assays using NUE in the presence of GST only or a second GST-labeled chlamydial protein, GST-CT529 (Fig. 6B). As expected, there was some weak methylation of GST itself but only when GST was in excess compared to GST-NUE (3∶1 or 5∶1 molar excess). To an even lesser degree, we saw methylation of GST-CT529. We also detected GST methylation in the GST-CT529 reactions due to the presence of GST alone contaminating our purified GST-CT529 (Fig. 6B, Coomassie stained gel). We conclude that the strong automethylation of GST-NUE occurs primarily within the NUE sequence.

### NUE automethylation regulates its activity towards histones

To determine which residue(s) is the target of automethylation, we conducted non-radioactive *in vitro* methyltransferase assays using NUE as both enzyme and substrate in the presence or absence of SAM. Comparison of these samples by mass-spectrophotometer revealed increased methylation in the presence of SAM of a peptide fragment containing two adjacent lysines, K209 and K210 (data not shown). The analysis could not distinguish between a di-methylation of one of these lysines or a single methylation event of both. We therefore generated mutants with both lysine residues mutated to either GG (NUEGG) or AA (NUEAA). We also used our C-terminal truncation mutant NUEΔ12 that lacks both K209 and K210 (see diagram Fig. 6C). We hypothesized that these mutations would not affect catalytic activity as the residues modified lie outside of the SET domain. Indeed, when we performed *in vitro* methylation assays using these mutants, each was able to automethylate to some degree demonstrating enzyme function is still intact (Fig. 6C). The residual automethylation is most likely attributed to the observation that the GST moiety itself can be methylated by NUE (Fig. 6B) and/or the possibility of alternative sites of methylation in the absence of the preferred residues. However, the decrease in signal compared to wild-type NUE suggests K209 and/or K210 serve as sites of automethylation. All three of the mutants demonstrated weaker histone methylation than the wild-type protein even when used at twice the concentration (Fig. 6C). These results suggest automethylation enhances NUE enzymatic activity towards its substrate as loss of two lysines targeted for methylation correlated with a decrease in histone methylation. Interestingly, the NUEGG mutant was slightly less impaired both in automethylation and towards histone substrate than the other two mutants. This suggests that this particular mutation overcomes to some extent the need for methyl groups on residues 209 and 210 to reach full enzymatic activity.

## Discussion

Bacterial effector proteins play crucial roles in pathogenicity. In this work, we have identified a new chlamydial effector protein, NUE, which contains a SET domain predictive of histone methyltransferase activity. We identified NUE as a novel TTS substrate of chlamydiae based on (i) the presence of NUE in the host cell nucleus during *C. trachomatis* infection, indicative that the protein is secreted outside the inclusion and (ii) the presence of a TTS signal in the N-terminal part of NUE from three different chlamydiae species. *In vitro* enzymatic assays confirmed the activity of NUE as a histone methyltransferase that modified mammalian histones H2B, H3 and H4. This is the first identified bacterial SET domain protein to directly enter the nucleus of the host cell and target mammalian histones.

Our observation that NUE methylates multiple histones is unusual but not unique. There are a growing number of mammalian examples in which a single histone methyltransferase has multiple histone and/or non-histone substrates. G9a is a well-characterized human SET domain protein that methylates both H3K9 and H3K27 [13]. A separate study identified G9a methylation of several non-histone proteins including chromodomain Y-like protein, widely interspaced zinc finger motifs protein, Cockayne syndrome group B protein and G9a itself (automethylation) [14]. Although not a SET domain protein, protein arginine *N*-methyltransferase 8 (PRMT8) is a histone methyltransferase that methylates both histones H2A and H4 and automethylates [29]. Interestingly, other pathogen effector proteins have been reported to have multiple mammalian targets. For example, *Listeria monocytogenes* induced dephosphorylation of histone H3 as well as deacetylation of both histones H3 and H4 [30]. In a separate study, the *Shigella flexneri* effector OspF was identified as a phosphatase that dephosphorylates two mitogen-activated protein kinases, ERK1 and p38, which ultimately leads to inhibition of histone H3 phosphorylation [31]. It is therefore entirely feasible that bacteria use their limited genome to target multiple substrates with a single effector. Studies are ongoing to determine the *in vivo* lysines targeted by NUE in order to analyze its specific effect on host cell chromatin.

Chlamydiae histone-like proteins Hc1 and Hc2 play a role in compacting DNA during the RB to EB transition [21], [22]. It was previously reported that Hc1 acts as a substrate of the *C. pneumoniae* NUE homolog in addition to histone H3 (other histones were not tested) [32]. To the contrary, we did not find methylation of Hc1 in our *in vitro* studies. Slight variations in protocol may contribute to the observed differences. Murata et al. used *C. pneumoniae* NUE with truncated Hc1 fragments as substrates. We used *C. trachomatis* with full-length Hc1. Additionally, we found NUE to be secreted by TTS, and substrates of TTS are often retained unfolded before secretion via association with a chaperone protein. If this is true for NUE, enzymatic activity within the bacteria would be unlikely.

In addition to its activity as a histone methyltransferase, NUE is an automethyltransferase. To date, there are two SET domain proteins reported to automethylate. G9a was shown to methylate its K239 residue [33]. Mutation analysis revealed the importance of this methylation event for G9a interaction with heterochromatin protein 1 (HP1) which therefore increased the function of the protein to act as a transcriptional repressor. In contrast, automethylation was shown to have little effect on the methyltransferase activity of Metnase but inhibited its decatenation activity [34]. Additionally, two non-SET domain HMTs, PRMT6 and PRMT8, automethylate. Automethylation of PRMT8 was shown to inhibit its function as a methyltransferase and the automethylation function of the fourth example, PRMT6, remains unexplored [29], [35]. Here we observed that mutation of the putative automethylation sites of NUE resulted in a decreased activity towards histone substrates. This suggests automethylation of NUE may enhance its methyltransferase activity perhaps by increasing its affinity for the target substrates.

One important question left opened by our study is the identity of the genes regulated by NUE translocation in the nucleus during *Chlamydia* infection. In the absence of a genetic system to manipulate *Chlamydia* it is difficult to answer. We have observed that NUE is expressed in low abundance during infection, a condition which is not reproduced by transfection approaches. Transcriptional studies have shown that, not surprisingly, numerous host genes are upregulated or downregulated during *Chlamydia* infection [36], [37]. One study that addresses global changes on host gene expression on a large time scale indicates that about 150 genes are up-regulated 36 hrs after infection of HeLa cells by *C. trachomatis* L2, against only about 75 genes 24 hrs post infection [38]. These data are in favor of the existence of mechanisms of control of gene expression by the bacteria that might be turned on only late in the infectious cycle, when NUE is expressed. While the cell is responding to dramatic changes triggered by infection, only some of its transcriptional response may result directly from NUE activity. Identification of NUE specific targets might come from the identification of its nuclear binding partner(s), which we are currently undertaking. We are also developing anti-NUE antibodies that would be suitable for chromatin immunoprecipitation approaches with infected cells.

SET domain proteins are present in all eukaryotes, and the few examples of such domains in bacterial proteins were initially interpreted to be the result of horizontal gene transfer. A more recent study favors the hypothesis that prokaryotic SET domain proteins evolved independently from a bacterial ancestor [39]. While our findings do not solve this intriguing question, we do show that the chlamydial SET domain protein evolved into a secreted protein targeting eukaryotic histones. Additionally, the ability of chlamydiae to sustain chronic infections presents an opportunity and motive for the bacteria to exert long-term epigenetic control of host cells. Any link between cleared chlamydial infection and dysregulation of genetic information due to epigenetic reprogramming, such as in cancer, will be challenging to establish as infections are often not diagnosed. Regardless of the long term implications, taking control of the host cell at the level of gene expression is certainly advantageous for a bacterium. Manipulating the histone code is one way to achieve this, and we can speculate that other pathogens that possess a SET domain protein may employ this strategy to sustain their own development.

## Materials and Methods

### Cell culture and chlamydial infection

HeLa and 3T3 cells (originally obtained from the ATCC) were cultured in DMEM culture medium supplemented with 5% fetal calf serum at 37°C and 5% CO_2_. *Chlamydia trachomatis* serovar L2 (originally obtained from ATCC) were harvested from infected cells as described previously with a few modifications [40]. Briefly, the bacteria were propagated in HeLa cells for 48 h. The cells were pelleted by centrifugation, resuspended in 10 ml of ice-cold SPG buffer (218 mM sucrose, 3.76 mM KH_2_PO_4_, 7.1 mM K_2_HPO_4_, 4.9 mM glutamate, pH 7.4) and then passed through a 22 G needle to lyse the mammalian cells. The resulting suspension was centrifuged at 500×g for 10 min to remove unbroken cells and nuclei. The new supernatant was centrifuged at 25000×g for 30 minutes at 4°C to collect the bacteria. The bacteria pellet was subsequently homogenized, resuspended in ice-cold SPG, aliquoted and stored at −80°C. Infection MOIs were determined by serial dilution of bacterial preps.

### Plasmids

The *E. coli* strain TG1 was used for plasmid constructions. *S. flexneri* and *E. coli* strains were grown in Luria-Bertani (LB). Full-length and C-terminal truncations of NUE were generated by PCR amplification of NUE from *C. trachomatis* D/UW-3/CX genomic DNA and were introduced into pDEST Gateway vectors (Invitrogen) by recombination as specified by the manufacturer's protocol. Amino acid substitution mutants were generated using Strategene Quickchange Mutgenesis Kit and verified by sequence analysis.

### Secretion assay and antibody production

Strains SF401 and SF620 are derivatives of M90T, the virulent, wild-type strain of *S. flexneri* 5, in which the *mxiD* and *ipaB* genes, respectively, have been inactivated [41]. The 5′ part of *nue* (including the first 29 codons for CT737, the first 27 codons for CPn0878 and the first 14 codons for CCA00889) were amplified by PCR and cloned in the puc19cya vector as described [42]. Secretion assays were performed on 30 ml of exponentially grown cultures as described previously [42]. Monoclonal antibody against Cya and polyclonal antibodies against CRP and IpaD were generously given by Drs. N. Guiso, A. Ullmann and C. Parsot, respectively (Institut Pasteur, Paris).

For NUE antibody production, the *nue* gene was amplified by PCR and cloned in the NcoI and KpnI sites of pQE-TriSystem vector (Qiagen). His-tagged NUE was grown in *E. coli*, purified using Ni-nitrilotriacetic acid agarose beads (Qiagen) and used as immunogen for the production of specific polyclonal antibodies in New Zealand White rabbits (Agro-Bio, La Ferté Saint-Aubin, France). The resulting rabbit anti-serum was then purified against GST-tagged NUE to obtain NUE antibody.

### Immunofluorescence

For immunofluorescence studies of transfected cells, 3T3 or HeLa cells were plated on glass coverslips and transfected using FuGene 6 (Roche) at a reagent to DNA ratio of 3∶1. After 24 or 48 h transfection, cells were washed once with PBS, fixed with 4% paraformaldehyde and permeabilized with 0.3% Triton X-100 for 5 min. Coverslips were then incubated with anti-FLAG (Sigma Aldrich) antibody in the presence of 0.1% bovine serum albumin, washed, incubated with Alexa Fluor 488 secondary antibody (Molecular Probes), washed repeatedly and mounted on glass slides using Mowiol containing 0.5 µg/ml Hoechst DNA stain.

### Nuclear isolation and Western blots

Infected (MOI of 1∶1) or transfected HeLa cells were washed once with PBS, pelleted and resuspended in buffer A (10 mM HEPES, pH 7.9, 10 mM KCl, 0.1 mM EDTA, 1 mM EGTA +protease inhibitor cocktail (Sigma, P8340)) for 10 min before addition of 0.2% NP-40 and passage through a 26 G syringe. Nuclei were pelleted at 800×g for 5 min, washed 1× with buffer A, resuspended in RIPA buffer (50 mM Tris, pH 7.4, 150 mM NaCl, 2 mM EDTA, 1% NP-40, 0.5% Na-deoxycholate, 0.1% SDS + protease inhibitor cocktail) for 30 min and centrifuged at 16000×g for 10 min. Protein concentrations were determined using the Bradford assay (Bio-Rad) and equal quantities from the cytosolic and nuclear fractions were loaded for analysis by western blot. Monoclonal anti-poly ADP ribose polymerase 1 (PARP1) antibody was purchased from Trevigen, monoclonal antibody against chlamydial EF-Tu was a kind gift from Y-X Zhang (Boston, USA) and polyclonal rabbit antibody against GDIβ was a kind gift from B. Goud (Institut Curie, France).

Separation of soluble nuclear fraction versus chromatin-associated proteins was done following the nuclear isolation protocol described above. Once isolated, nuclei were resuspended in buffer B (3 mM EDTA, 0.2 mM EGTA, 1 mM DTT) for 30 min and centrifuged at 1700×g for 5 min. Supernatants were collected as soluble nuclear fraction and the chromatin pellet was washed 2 times in buffer B before resuspension in 1× sample buffer. All samples were boiled for 5 min prior to loading for separation on several identically loaded SDS-PAGE gels. All gels were then transferred to a PVDF membrane, blocked in 5% BSA for 1 h and incubated with appropriate antibodies. Rabbit polyclonal anti-Suv39H1 antibodies were from Upstate (#07-550), anti-Histone H3 antibodies from Sigma (H0164).

### Protein purification and HMT assay

GST-tagged proteins (NUE or NUE mutants) were produced in *E. coli* grown to log phase and induced with 0.5 mM isopropyl-beta-D-thiogalactopyranoside (IPTG) overnight at 20°C. Bacteria were centrifuged at 3800×g for 15 min at 4°C, resuspended in lysis buffer (50 mM Tris pH 7.5, 100 mM NaCl, 1 mM EDTA, 10% glycerol, 0.01% Triton X-100, 1 mM PMSF) for 30 min, sonicated and centrifuged at 17200×g for 20 min at 4°C. Supernatants were incubated with glutathione sepharose beads (Pierce) for 1 h at 4°C, washed 3 times and protein was eluted from the beads using 20, 50 and 100 mM reduced glutathione (pH adjusted to 8). Protein containing fractions (determined by Coomassie blue staining) were pooled, concentrated and measured by Bradford assay to determine final protein concentration. His-tagged Hc1 (*hctA* plasmid was the generous gift of Dr. Ming Tan, University of California Irvine) was produced in *E. coli* grown to log phase and induced with 0.2% L-arabinose for 3 h. Bacteria were centrifuged, resuspended in lysis buffer (10 mM Tris pH 8, 300 mM NaCl, 2 mM imidazole plus protease inhibitors) and sonicated to ensure lysis. After centrifugation, supernatants were incubated with Ni-NTA agarose (Qiagen), washed and protein was eluted with 10, 50, 250 or 500 mM imidazole. Eluate was then dialysed with storage buffer (10 mM Tris pH 8, 10 mM MgCl2, 0.1 mM EDTA, 100 mM NaCl), concentrated and protein concentration was measured by Bradford assay (Bio-Rad).

HMT assays were performed using 4 µg of recombinant NUE and/or NUE mutants in equal or molar excess. Assays were carried out in 25 µL of assay buffer (50 mM Tris pH 8, 20 mM KCl, 250 mM sucrose, 10 mM MgCl2, 1 mM DTT) plus or minus core histones (Sigma Aldrich, H9250) or individual recombinant histones (New England Biolabs) and S-[methyl-C14]-adenosyl-L-methionine (PerkinElmer) for 1 h at 30°C. Samples were boiled for 5 min before migration on 15% SDS-PAGE gels. All gels were stained with Coomassie blue to visualize protein loading before drying and 24 h exposure for analysis of radioisotope incorporation. Control proteins GST-CT529 and GST only were purified from *E. coli* as described above for GST-NUE.

For mass-spectrophotometric analysis, after incubation of GST-CT737 with SAM and in-gel digestion of the reaction product with trypsin, the lysine residues and the N-termini were labeled with nicotinic acid and then subjected to small scale reverse phase chromatography. The sample was then eluted with a modified step gradient from 10–65% acetonitrile and each fraction was spotted onto a MALDI plate. The spectra were acquired from each plate and analyzed using PROFOUND and PEPMAP programs to identify potentially methylated peptides. MS/MS (fragmentation) spectra were taken of the top 15 peptides per fraction and were searched using MASCOT or X!Tandem.

### Accession numbers/ID numbers for proteins mentioned in the text

NUE in *C. caviae* – NP_829751.1

NUE in *C. pneumoniae* – NP_300935.1

NUE in *C. abortus* – YP_220244.1

NUE in *C. trachomatis* serovar D – NP_220256.1

NUE in *C. muridarium* – NP_296494.1

NUE in *C. felis* – YP_515042.1

SET8 – AAM47033

MLL4 – AAH09337.2

G9a – CAA49491

Suv39H1 – CAG46546.1

Hc1 - AAA23129.1

## Supporting Information

Figure S1 Purified NUE antibody is protein-specific. NUE antibody was generated and purified as described in Material and Methods. Lysates from non-infected HeLa cells (“−”) or cells infected with *C. trachomatis* (“+”) for 48 hours were loaded on a SDS-PAGE gel, transferred to a PVDF membrane and probed with anti-NUE (1^st^ panel), anti-NUE in the presence of 5 µg/ml NUE purified protein (2^nd^ panel) or anti-NUE in the presence of 5 µg/ml CT671 purified protein (3^rd^ panel), an irrelevant protein purified in the same conditions as NUE. The boxed portion of the gel indicates the predicted location of NUE (predicted molecular weight is 25 kDa).
(0.65 MB TIF)
