# Supplementary figures and images for: Histone Methylation by NUE, a Novel Nuclear Effector of the Intracellular Pathogen Chlamydia trachomatis
Source: PLoS Pathog. 2010 Jul 15;6(7):e1000995. doi: 10.1371/journal.ppat.1000995 (PMC2904774; doi:10.1371/journal.ppat.1000995)

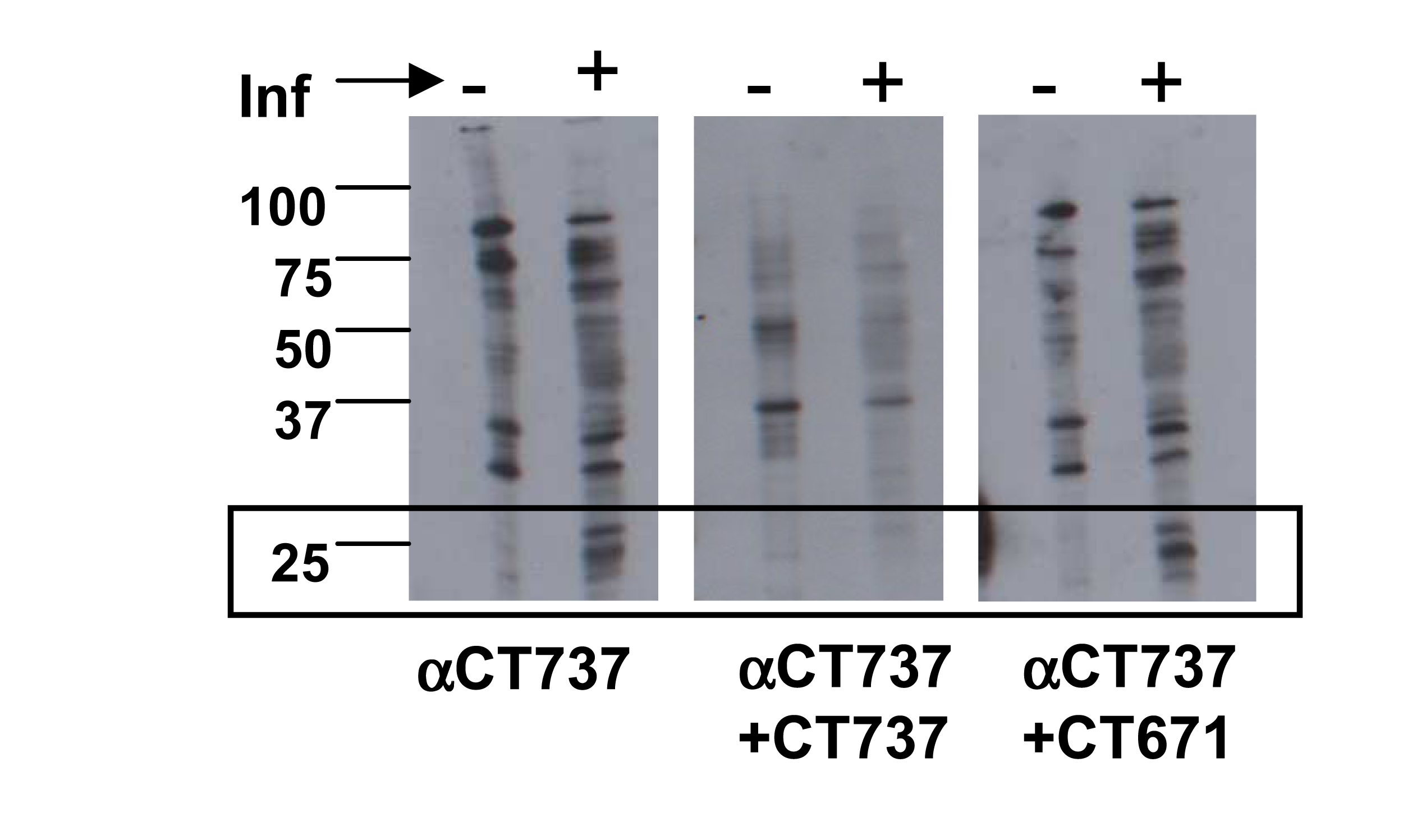

Supplement: Figure S1 — Purified NUE antibody is protein-specific. NUE antibody was generated and purified as described in Material and Methods. Lysates from non-infected HeLa cells (“−”) or cells infected with C. trachomatis (“+”) for 48 hours were loaded on a SDS-PAGE gel, transferred to a PVDF membrane and probed with anti-NUE (1st panel), anti-NUE in the presence of 5 µg/ml NUE purified protein (2nd panel) or anti-NUE in the presence of 5 µg/ml CT671 purified protein (3rd panel), an irrelevant protein purified in the same conditions as NUE. The boxed portion of the gel indicates the predicted location of NUE (predicted molecular weight is 25 kDa). (0.65 MB TIF) [file ppat.1000995.s001.tif]
